# Neuro-Genetic Optimization of the Diffuser Elements for Applications in a Valveless Diaphragm Micropumps System

**DOI:** 10.3390/s90907481

**Published:** 2009-09-18

**Authors:** Hing Wah Lee, Ishak Hj Abdul Azid

**Affiliations:** 1 Microfludics and BioMEMS, MIMOS BERHAD, Technology Park Malaysia, 57000 Kuala Lumpur, Malaysia; 2 Department of Mechanical Engineering, Universiti Sains Malaysia, 11800 Penang, Malaysia; E-Mail: ishak@eng.usm.my

**Keywords:** optimization, artificial neural network, genetic algorithms, diffuser, valveless micropump

## Abstract

In this study, a hybridized neuro-genetic optimization methodology realized by embedding numerical simulations trained artificial neural networks (ANN) into a genetic algorithm (GA) is used to optimize the flow rectification efficiency of the diffuser element for a valveless diaphragm micropump application. A higher efficiency ratio of the diffuser element consequently yields a higher flow rate for the micropump. For that purpose, optimization of the diffuser element is essential to determine the maximum pumping rate that the micropump is able to generate. Numerical simulations are initially carried out using CoventorWare® to analyze the effects of varying parameters such as diffuser angle, Reynolds number and aspect ratio on the volumetric flow rate of the micropump. A limited range of simulation results will then be used to train the neural network via back-propagation algorithm and optimization process commence subsequently by embedding the trained ANN results as a fitness function into GA. The objective of the optimization is to maximize the efficiency ratio of the diffuser element for the range of parameters investigated. The optimized efficiency ratio obtained from the neuro-genetic optimization is 1.38, which is higher than any of the maximum efficiency ratio attained from the overall parametric studies, establishing the superiority of the optimization method.

## 1. Introduction

In recent developments, micropumps utilizing piezoelectric actuation have been commonly employed for directing the fluid purposes especially in BioMEMS and microfluidic systems [1–6]. One of the essential features of micropump is the ability to direct the fluid flow as to flow in only one direction and this could be enhanced with the introduction of a check valve. It has been shown that micropumps having the best performance in terms of pressure/flow characteristics are those utilizing passive-types of check valve [7] or those with valveless diffuser elements [8–11]. However, passive check valves incorporated in micropumps induce difficulties such as clogging and sedimentation and have a rather complicated design [7]. Therefore, they are mostly deemed unsuitable for miniaturization purposes.

On the other hand, the earliest concept of valveless micropumps incorporating diffuser elements have been developed [8,9] with the hopes of eliminating the additional voltage requirement for operating the check valves while at the same time, improve the reliability aspect of the micropump since no mechanical moving parts are involved. Nevertheless, it was not until 1997 that the first valveless diffuser micropump in silicon was introduced by Olsson *et al.* [11], which subsequently led to great interest on diffuser elements for applications in micropumps.

**Figure 1.**
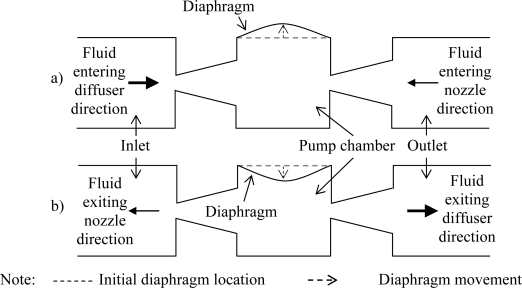
Working principle of a typical valveless micropump utilizing diffuser element during (a) suction mode (b) supply mode.

Generally, valveless diffuser micropumps are driven by a piezoelectric element bonded to a flexible diaphragm with no additional moving parts. Application of voltage on the piezoelectric element induces deformation on the diaphragm which creates displacement in the vertical direction (Figure 1) and generates pressure head inside the pump chamber. The ability of the micropump to direct the working fluid inside the chamber is based upon the flow resistance in the diffuser elements.

The working principle of the diffuser elements in a valveless micropump is schematically shown in Figure 1. In the “suction mode”, the diaphragm moves vertically upwards increasing the chamber volume and causes reduction in the chamber pressure. Pressure difference between the pump chamber and inlet/outlet enables the working fluid to be sucked into the pump chamber from both the inlet and outlet. At this instance, fluid will enter the pump chamber from the inlet through the diffuser direction while at the outlet, fluid will enter through the diffuser element at the nozzle direction instead. For the “pumping mode”, the reverse phenomenon occurs. The rate of fluid flow entering/exiting the chamber from/to the inlet and outlet is dependent on the design of the diffuser element where the effectiveness of the flow rectification of the micropump can be gauged upon the net flow of the fluid from the inlet to the outlet (which is the desired flow direction) during pumping. Hence optimization for the design of the diffuser element is of extreme importance in order to develop micropumps capable of operating at maximum efficiency.

Investigation on the effects of the diffuser element on the performance of valveless micropumps have been analyzed previously by simulating the diffuser model either by using commercially available numerical simulation software [12] or through analytical works [13–20]. However, none of these analyses focused on the design optimization of the diffuser element to generate micropump with the maximum net flow rate considering the working conditions or the required specifications of the micropump.

The purpose of this paper will be to present a neuro-genetic methodology to simulate and optimize the performance of the diffuser elements for applications in valveless diaphragm micropumps. The optimization methodology begins by simulating the diffuser model using commercially available numerical simulation software package, CoventorWare® to study the performance of the diffuser element under different working conditions and geometrical parameters. The simulation results obtained will be utilized for training the artificial neural networks (ANN) model.

An artificial neural network (ANN) is based on the working process of human brain in decision making. It is categorized as an artificial intelligence method and has been applied in many different fields such as control [21], finance, aerospace, industrial and manufacturing [22,23]. The typical neural network consists of sets of inputs, sets of outputs and weighting functions. By knowing the input values, the output can be predicted. In other words, the network is defined to correlate between input and output by training the network with available data, such as results from numerical simulations. Once trained, the network can then be fed with any unknown input and is expected to predict the output (in this work, the diffuser efficiency ratio) with a high level of accuracy.

In order to perform optimization, direct associations between ANN and the optimization tool is required. Genetic algorithm (GA), which is also categorized as an artificial intelligence method, offers compatibility with ANN since GA would be able to find the global optimum in the local parametric search space provided by ANN. In view of that, the trained ANN is embedded as a fitness function into GA where the combined artificial neural network-genetic algorithm; hence the term “neuro-genetic”; will be used in sequence as a tool for search and optimization purposes. GA was the desired optimization techniques as both ANN and GA can be easily modeled and integrated in Matlab® since toolbox for both of these techniques is available as standard. Generally, GA is a robust adaptive search method based on Darwinian principles of natural selection, survival of the fittest and natural genetics. It combines survival of the fittest among string structures with a structured yet randomized information exchange to form a search algorithm with some of the innovative flair of human search [24]. GA has been widely used for optimization purposes in microelectronic problems as shown in previous works by Man *et al*. [25], Arunasalam *et al.* [26] and Jeevan *et al.* [27], while a more detailed description of GA can be found texts written by Goldberg [24], Mitchell [28] and Houck *et al.* [29].

In this work, the purpose of the neuro-genetic optimization was to find the maximum efficiency ratio of diffuser element based on the diffuser angle, Reynolds number and aspect ratio under the specified working conditions and geometrical parameters of the micropump.

## 2. Design and Modeling

### 2.1. Theoretical and Dimensional Analysis of the Diffuser Element

**Figure 2.**
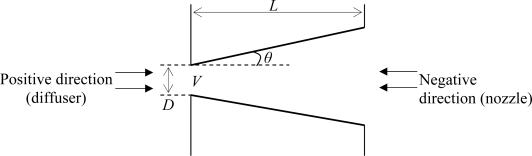
Fluid flow in a diffuser element.

A schematic of the typical diffuser element is shown in Figure 2, where fluid flow in the positive direction usually depicts flow through the diffuser while fluid will be considered flowing through the nozzle for the negative direction.

Generally the effectiveness of the diffuser element for applications in valveless micropump is gauged through the flow rectification efficiency. The flow rectification efficiency is the measure of the ability of the pump to direct the flow in one preferential direction and can be defined as the ratio of the micropump net flow rate, *Q_net_* to the rate of displaced volume, *V̇* as given by:

(1) $$\gamma =\frac{Q_{\mathrm{net}}}{\overset{•}{V}}$$

The pressure loss for the diffuser element at both the diffuser and nozzle direction can be represented in terms of the pressure loss coefficient, *ξ* as:

(2) $$\Delta P_{d}=\xi_{d}\;\frac{1}{2}\;\rho {\left(\frac{Q_{d}}{A}\right)}^{2}$$

(3) $$\Delta P_{n}=\xi_{n}\;\frac{1}{2}\;\rho {\left(\frac{Q_{n}}{A}\right)}^{2}$$

where *ρ* is the fluid density, *Q* the mean volumetric flow rate at the throat of the diffuser element and *A* the cross-sectional area of the throat while the subscripts *d* and *n* denote the diffuser and nozzle direction, respectively.

Based on the geometrical relationship and continuity equation of fluid flow, the rate of displaced volume satisfy:

(4) $$\overset{•}{V}=Q_{d}+Q_{n}$$

Hence solving Equations (1), (2), (3) and (4) simultaneously, the rectification parameter can be obtained as the ratio of the net flow rate to the rate of displaced volume, as given by:

(5) $$\begin{array}{ll} \gamma & =\frac{Q_{\mathrm{net}}}{\overset{•}{V}}\\ & =\frac{Q_{d}-Q_{n}}{Q_{d}+Q_{n}}\\ & =\frac{\sqrt{\xi_{n}}-\sqrt{\xi_{d}}}{\sqrt{\xi_{n}}+\sqrt{\xi_{d}}} \end{array}$$

For the same opening angle *θ* for the diffuser element, the pressure loss coefficient at the negative direction is higher than that at the diffuser positive direction. Hence the diffuser element efficiency ratio can be expressed as:

(6) $$\eta =\frac{\xi_{n}}{\xi_{d}}$$

Utilizing expression from Equation (6), Equation (5) can now be written in a dimensionless form as:

(7) $$\gamma =\frac{\sqrt{\eta }-1}{\sqrt{\eta }+1}$$

The dimensional analysis for the diffuser element using Buckingham ∏ theorem presented by Ahmadian and Mehrabian [18] showed that for steady state conditions, the diffuser element efficiency ratio (and subsequently the rectification parameter) depends upon the geometrical parameters and Reynolds number. The optimization task is stated as:

(8) η=f(Re,AR,θ)

where Re is the Reynolds number given by Re = *ρVD*/*μ* with *V* as the average throat velocity and *μ* as the fluid dynamic viscosity, *AR* is the diffuser aspect ratio given by I = *L*/*D* and *θ* is the diffuser opening angle.

### 2.2. Numerical Simulation

**Figure 3.**
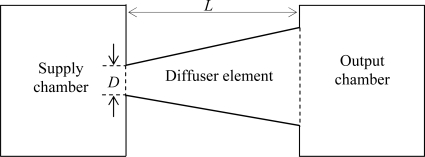
Simplified model of the valveless micropump.

In this work, a simplified model of the valveless micropump has been proposed to evaluate the performance of the diffuser element. The numerical model consists of a supply chamber, an output chamber and a diffuser element as shown in Figure 3. It should be noted that only planar diffuser element is considered. Simulations will be performed using MemCFD™ module under CoventorWare® with FLUENT™ as the finite volume solver.

**Figure 4.**
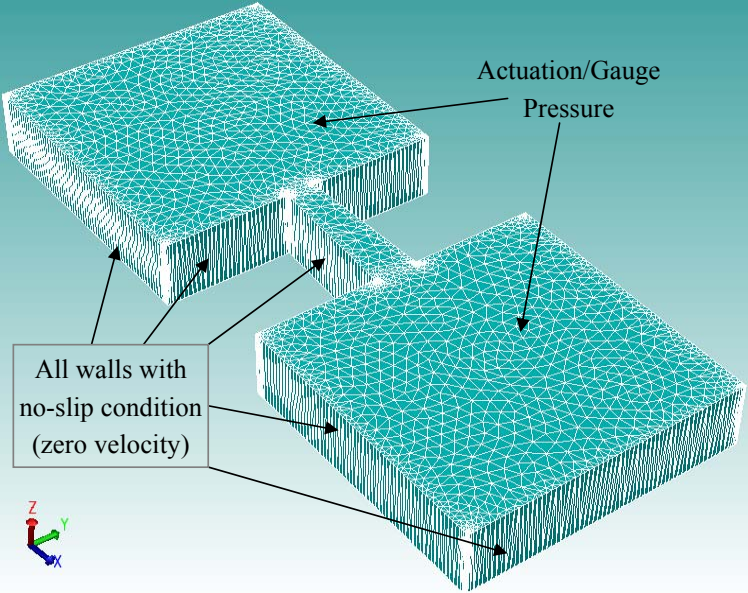
Meshed model of the valveless micropump with applied boundary conditions.

In order to ease computational demands, only a 2-D simulation of the micropump was carried out. This could be achieved in CoventorWare® by creating only a single element through the thickness of the micropump with mesh refinement at the edges. Tetrahedrons mesh type will be used and mesh sensitivity analysis will be conducted for preliminary simulation to determine the minimum element size required to ensure that the simulation results obtained will be independent of the meshing densities. This could be achieved by refining the mesh until the change in the simulation results is within 1% as shown in Table 1. Consequently, the element size will be set to 50 for all subsequent simulations. All the walls of the micropump; except the boundaries to be applied with pressure; will be attached with zero velocity to represent the no-slip conditions of the flow. The top surface of the supply and outlet chamber will be applied with either the actuation or the gauge pressure based on the fluid flow direction. For flow through the diffuser (positive) direction, the top surface of the supply and outlet chamber will be applied with the actuation and the gauge pressure respectively, and vice versa for flow through the nozzle direction. Figure 4 shows the meshed model of the valveless micropump along with the boundary conditions defined in the simulation.

All the simulations use water as the working liquid, thereby limiting the problem to incompressible flow. Actuation pressures ranging from 1 kPa to 20 kPa applied on the supply chamber and fluid flow through the diffuser element will be considered as laminar since the Reynolds numbers is expected at below 500 based on previous studies of the diffuser element [16,17]. The thickness of the diffuser element has been fixed at 150 μm based on the standard thickness of SU8-100 (MicroChem Corp) negative photoresist to be used for patterning the diffuser structures. Parametric studies will be conducted for flow through the diffuser element using range of parameters provided by Table 2. This is achieved by keeping the other parameters constant using the basic design value whenever the parameter of interest is varied. Results for the average throat velocity and pressure loss obtained from the steady-state analysis will be used to calculate the flow Reynolds number, pressure loss coefficient and subsequently, both the efficiency ratio and rectification parameter of the diffuser element.

### 2.3. Neuro-Genetic Optimization

**Figure 5.**
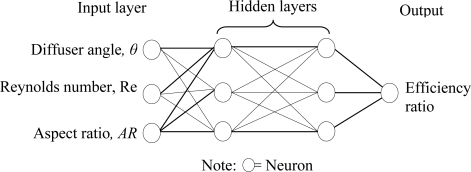
Illustration of the artificial neural network model.

Simulation results obtained from CoventorWare® simulations will be used to train the ANN model so that the diffuser element efficiency ratio can be predicted for different diffuser angle, Reynolds number and aspect ratio. The artificial neural network used in the present study is shown in Figure 5 where there are three neurons in the input layer and one neuron in the output layer. The architecture used for the artificial neural network is the feed-forward multilayer perceptron neural network while the training method implemented is the back-propagation algorithm with two hidden layers. Table 3 shows the details of the ANN model used in this work where the overall simulation has been accomplished using MATLAB® R2008a.

It should be noted that from the range of parameters simulated in CoventorWare®, there will be five sets of data for each category of parameters that will not be used as training inputs to the ANN. This is because simulation data that were not trained will be used instead to verify the accuracy of the ANN results. This is performed by comparing the predicted ANN results to the CoventorWare® simulation results. Once the ANN results are validated, a well trained ANN will be established. At this stage any new sets of values for parameters such as diffuser angle, Reynolds number and aspect ratio that are fed into the ANN model will, upon simulation, generate prediction for the efficiency ratio with high level of accuracy.

After completing the training process, the trained ANN will then be embedded into genetic algorithms (GA) for the search and optimization purpose. Simulation for the GA has been performed using a self-developed code in Matlab® through modification to the Genetic Algorithm Toolbox (GAOT) [28] to enable direct interaction with ANN. The objective of the optimization is to maximize the efficiency ratio for the diffuser element. GA will generate initial population for parameters such as Re, *AR* and *θ* where these data are subsequently fed into the trained ANN algorithm as input variables to predict the efficiency ratio of the diffuser element. The predicted efficiency ratio will undergo an evaluation process by GA and is assigned a fitness score which is then measured, recorded, ranked and compared with previous iteration results. The top grade chromosomes (highest value of fitness score) from the evaluation process will be selected and allowed to reproduce with other individuals in the population where they will undergo the crossover and mutation processes using parameters given by Tables 4 and 5. These two processes produce new individuals that will become the new population of solutions for the next generation. The new individuals share some traits and features from the parent. Members of the population with a low fitness value will be discarded and are unlikely to be selected for the next evolution process. The population of the newly recombined and mutated chromosomes will become the new input parameters for the trained ANN which subsequently predicts the efficiency ratio for a new cycle of iteration in GA. The entire process of evaluation and reproduction then continues until either the population converges to an optimal solution for the problem or the stopping criterion has been met. Finally, the converged result obtained will yield the maximum efficiency ratio of the diffuser element for the range of parameters considered.

## 3. Results and Discussion

**Figure 6.**
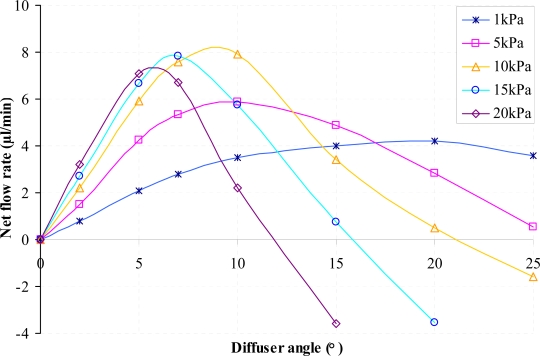
Net flow rate at the throat of diffuser element for variation in diffuser angle at different actuation pressure (*AR* = 20).

**Figure 7.**
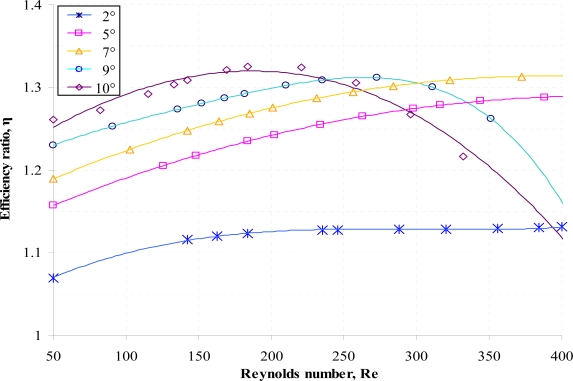
Efficiency ratio of the diffuser element for variation in Reynolds number at different diffuser angle (*AR* = 20).

**Figure 8.**
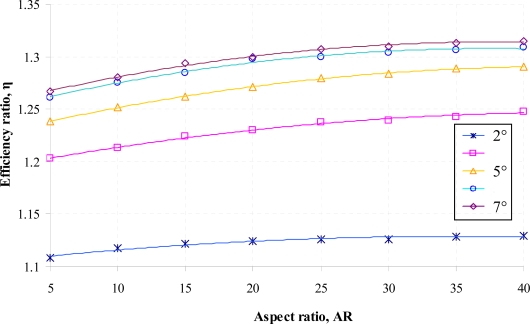
Efficiency ratio of the diffuser element for variation in aspect ratio at different diffuser angle (Re = 200) at a constant actuation pressure of 10 kPa.

Numerical simulations for both the positive and negative direction of flow for the diffuser element have been conducted using the range of parameters given earlier in Table 1. Results of the volumetric net flow rate at the throat of the diffuser element obtained from CoventorWare® simulations for different diffuser angle is presented in Figure 6 while Figure 7 and Figure 8 shows the efficiency ratio of the diffuser element for variations in Reynolds number and aspect ratio.

The net flow rate shows the effective fluid volume flowing through the diffuser element in the desired direction (positive direction). From Figure 6 it can be seen that for the respective actuation pressure, an increase of the diffuser angle causes the net flow rate of the diffuser element to increase until reaching a maximum value. Any subsequent increase of the diffuser angle after the maximum net flow rate is reached will results in reduction of the net flow rate until the state where the net flow rate will become zero. At this instance, any further increment of the diffuser angle will instead create backflow where the net flow rate will be from the negative direction. It is apparent that there is an instance where the optimized net flow rate could be achieved at a specific diffuser angle. For cases where the net flow rate is negative, the volumetric flow rate is higher in the nozzle direction as compared to the diffuser direction due to the higher pressure loss coefficient in the diffuser direction. At this stage, net amount of fluid will be instead sucked into the supply chamber each pumping cycle.

Similar characteristics can be observed for variations in Reynolds number where there exists an optimized maximum efficiency ratio for a given diffuser angle as shown in Figure 7. At higher Re, reduction in the efficiency ratio is imminent as flow separation; the phenomenon where some parts of the flow are actually going in a direction opposite to the bulk flow direction; is higher through the diffuser direction. This is due to the fact that at higher Re, the free stream flow velocity will be higher and will consequently be reduced in a rapid manner due to effect of the adverse pressure gradient present in the diffuser element. The sudden reduction of the free stream flow velocity resulted in the boundary layer unable to be sustained without separating from the wall. At this instance, flow separation will occur where another region of flow, in the opposite direction to the bulk flow direction, will be created near the wall. Meanwhile, pressure gradient inherent at the nozzle direction decreases with the flow direction due to the increasing free stream flow velocity. Hence at increasing Reynolds number, flow separation; which directly contributes to the pressure loss coefficient; increases more rapidly in the diffuser direction as compared to the nozzle direction. It is also apparent that reduction in the efficiency ratio for high Re is more significant at higher diffuser angle since expansion of the diffuser angle promotes more flow separation due to the sudden increase of the flow area which results in the reduction of the free stream flow velocity and consequently increases the adverse pressure gradient. In view of this, the overall pressure loss coefficient of the flow along the diffuser element will be dominated by the occurrence of the flow separation at higher diffuser angle.

The effect of the diffuser element aspect ratio on the efficiency ratio is given in Figure 8. As can be observed in the figure, there is a relatively small influence of the diffuser element aspect ratio on the efficiency ratio although generally the efficiency ratio increases when higher aspect ratio of the diffuser element is used. This is due to the fact that as the length increases, a small change of velocity in the diffuser element will be encountered resulting in a small amount of additional pressure recovery for flow through the diffuser direction. Hence a lower pressure loss coefficient at the diffuser direction, *ξ_d_* will be generated for the diffuser element. These findings concur well with the experimental results presented by Olsson *et al.* [10,11].

Following results obtained, it can be ascertained that there are distinctive features for the influence of each parameters (diffuser angle, Reynolds number and aspect ratio) on the efficiency ratio of the diffuser element. Since mathematical modeling relating the efficiency ratio to *θ*, Re and *AR* is unknown, optimization could only be achieved by utilizing artificial intelligence tool which is capable of correlating the design parameters to the efficiency ratio with ease. For that purpose, the neuro-genetic optimization method has been performed to find the maximum efficiency ratio for the diffuser element under the range of parameters investigated.

**Figure 9.**
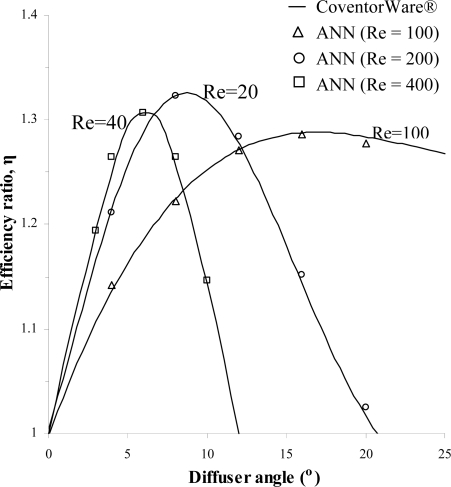
Comparison of efficiency ratio obtained from CoventorWare® simulations with ANN predictions for variations in diffuser angle at different Re numbers.

In order to assess the accuracy of the ANN predictions, validation has been made by comparing results for the efficiency ratio of the untrained numerical simulations with the ANN predictions. Figure 9 shows comparison of the CoventorWare® simulations results with ANN predictions for the efficiency ratio obtained from variations of the diffuser angle at different Reynolds number. Additional comparison of results between CoventorWare® simulations and ANN predictions are presented in Table 6. As mentioned earlier, five sets of data from each category of parameters from the numerical simulations that were not used for training in ANN will be presented for comparison purpose. From the results of Figure 9 and Table 6, it can be ascertain that the trained ANN was able to predict accurately the efficiency ratio of the diffuser element for the range of design parameters investigated. The percentage of errors from the ANN predictions is less than 2% while negligible for some cases, establishing the ANN predictions superiority.

**Figure 10.**
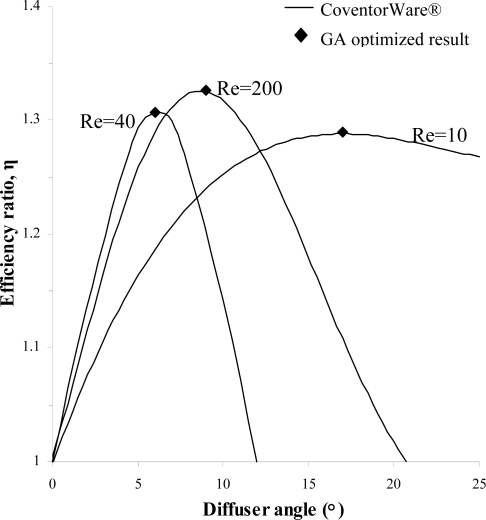
Maximum efficiency ratio obtained using GA for variations in *θ* at different Re.

Once a well-trained ANN is found, it is then embedded as a fitness function into genetic algorithms (GA) for optimization purposes. The optimization analysis has been performed to find the maximum efficiency ratio from the range of parameters available for selection in the trained ANN. Figure 10 shows an example of utilizing GA to find the maximum efficiency ratio for variations in diffuser angle at fixed Reynolds number of 100, 200 and 400. The maximum efficiency ratios obtained along with the corresponding diffuser angle for each Reynolds number are shown in Table 7 where it should be noted that only integers are used for parameters associated with the diffuser angle.

For the range of parameters indicated earlier in Table 1 that have been considered for simulations in CoventorWare® and used for training the ANN, the maximum efficiency ratio attainable is 1.38 as optimized by the neuro-genetic methodology. The optimized maximum efficiency ratio achieved is the highest obtained thus far when compared against results from the overall parametric studies. This is due to the fact that the neuro-genetic optimization will be able to take into account all the possible combinations of parameters to generate the predicted output which are then compared until the highest efficiency ratio is found. However, it should be made clear that the highest efficiency ratio produced is only valid for application of the diffuser element within the range of parameters investigated. Another point to note is that in actual application, the range of Reynolds number varies according to flow condition. Hence the expected range of Reynolds number for the flow in the diffuser element should be estimated in advance so that the optimized parameters can be chosen based on the perceived application. Table 8 shows parameters associated with the maximum efficiency ratio obtained while Table 9 shows the five highest efficiency ratios generated by the neuro-genetic optimization along with the corresponding parameters. Results from the present study clearly indicate that a well trained ANN combined with GA can be used to optimize the efficiency ratio of the diffuser element with confidence. The neuro-genetic optimization methodology is able to predict the maximum efficiency ratio for the range of parameters under consideration without requiring the actual mathematical model governing the behavior of the fluid flow across the diffuser element to be defined. Additionally parametric studies can be conducted with ease through ANN simulations using only few numerical simulation results as the training inputs, eliminating the needs for extensive remodeling of the numerical model.

## 4. Conclusions

The efficiency ratio of the diffuser element has been successfully optimized for valveless diaphragm micropump applications. The optimization process has been realized using a hybridized neuro-genetic optimization methodology by embedding numerical simulations trained artificial neural networks (ANN) into genetic algorithms (GA). The fluid flow behavior through the diffuser element has been initially simulated using CoventorWare® software for different diffuser angles, Reynolds numbers and aspect ratios where results obtained have been used for training the ANN model via the back-propagation method. The trained ANN is a superior tool when utilized to conduct parametric studies where it has been shown that predictions with errors of less than 2% were generated. The trained ANN, combined with GA to form the neuro-genetic tool, predicted that the maximum efficiency ratio is 1.38 for the range of parameters investigated. The predicted efficiency ratio is higher than the maximum efficiency ratio attained from the overall parametric studies, establishing the optimization method superiority.

## Figures and Tables

**Table 1.** Mesh sensitivity analysis for flow at diffuser direction (Diffuser angle = 5°; *AR* = 20).

**Element size**	**Number of elements**	**Flow rate (×10^3^μl/s)**	**%change**	**Simulation time (min)**
500	3921	9.9328	-	12
200	6371	9.7247	2.139912	23
100	7757	9.4892	2.481769	41
50	13523	9.3241	1.77068	52
30	55720	9.2309	1.009652	97
20	85237	9.2309	0	138

**Table 2.** Range of parameters simulated in CoventorWare®.

**Parameters**	**Range**	**Basic**
Throat width, *D* (μm)	50–100	100
Diffuser length, *L* (μm)	*Depends upon aspect ratio	2000
Thickness (μm)	–	150
Aspect ratio, *AR*	5–40	20
Chamber width, *W* (μm)	–	5000
Diffuser angle, *θ* (°)	2–25	2, 5, 7, 9, 10
Actuation pressure, *P* (kPa)	1–20	1, 5, 10, 15, 20

**Table 3.** Architecture of the ANN.

**Parameters**	**Type/Value**
Architecture	Feed-forward
Training algorithm	Back-propagation
Transfer function	All logsig
Hidden layer and neurons	2 hidden layer with 3 neurons each
Maximum epoch	1000
Learning rate	0.000001
Sum Square Error (SSE)	1e–5

**Table 4.** Crossovers parameters.

**Type**	**Parameters**
Arithmetic	4
Heuristic	[2 3]
Simple	4

**Table 5.** Mutations parameters.

**Type**	**Parameters**
Boundary	3
Multi Non-Uniform	[3 10 1]
Non-Uniform	[3 10 1]
Uniform	3

**Table 6.** Comparison of efficiency ratio results obtained from CoventorWare® simulations with ANN prediction for different parameters (Basic design values: *θ* = 5°; Re = 200; AR = 20).

**Parameters**	**Efficiency ratio**		**Variations (%)**
	**CoventorWare®**	**ANN**	
θ = 4°	1.2153	1.2197	0.358
θ = 8°	1.3236	1.3241	0.042
θ = 12°	1.2776	1.2711	−0.504
θ = 16°	1.1432	1.1345	−0.766
θ = 20°	1.0172	1.0093	−0.771
Re = 100	1.1904	1.1986	0.684
Re = 150	1.2075	1.2096	0.174
Re = 250	1.2553	1.2620	0.534
Re = 300	1.2750	1.2643	−0.840
Re = 350	1.2844	1.3016	1.340
AR = 10	1.2135	1.2364	1.887
AR = 15	1.2240	1.2266	0.213
AR = 25	1.2397	1.2512	0.928
AR = 30	1.2390	1.2260	−1.050
AR = 35	1.2430	1.2440	0.081

**Table 7.** Maximum efficiency ratio obtained using GA for different diffuser angle.

**Reynolds number, Re**	**Maximum efficiency ratio, *η***	**Optimized diffuser angle, *θ* (°)**
100	1.290	17
200	1.326	9
400	1.307	6

**Table 8.** Parameters associated with the diffuser element to obtain the highest efficiency ratio.

**Parameter**	**Value**
Diffuser angle, *θ* (°)	8
Reynolds number, Re	240
Aspect ratio, *AR*	40

Maximum efficiency ratio, *η*	1.38

**Table 9.** Five highest efficiency ratios obtained along with the corresponding parameters generated using neuro-genetic optimization.

**Diffuser angle, *θ* (°)**	**Reynolds number, Re**	**Aspect ratio, *AR***	**Optimized efficiency ratio, *η***
8	220	40	1.38
9	220	40	1.36
7	220	40	1.35
9	200	40	1.35
8	210	40	1.34

## References

[1] Zegerle R., Urlich J., Kluge S., Richter M., Richter A. (1995). A Bidirectional Silicon Micropump. Sens. Actuat. A: Phys.

[2] Gerlach T., Wurmus H. (1995). Working Principle and Performance of the Dynamic Micropump. Sens. Actuat. A: Phys.

[3] Ulises F.G., Walied A.M. (2002). Simulation of MEMS Piezoelectric Micropump for Biomedical Applications. ASME Int. Mech. Eng. Congr. Expos.

[4] Teymoori M.M., Abbaspour-Sani E. (2005). Design and Simulation of a Novel Electrostatic Peristaltic Micromachined Pump for Drug Delivery Applications. Sens. Actuat. A: Phys.

[5] Lan W.P., Chang J.S., Wu K.C., Shih Y.C. Simulation of Valveless Micropump and Mode Analysis.

[6] Fan B., Song G., Hussain F. (2005). Simulation of a Piezoelectrically Actuated Valveless Micropump. J. Smart Mater. Struct.

[7] Gravesen P., Branebjerg J., Jensen O.S. (1993). Microfluidics: A Review. J. Micromech. Microeng.

[8] Stemme E., Stemme G. (1993). A Valve-less Diffuser/Nozzle Based Fluid Pump. Sens. Actuat. A: Phys.

[9] Olsson A., Stemme G., Stemme E. (1995). A Valve-Less Planar Fluid Pump with Two Chambers. Sens. Actuat. A: Phys.

[10] Olsson A., Stemme G., Stemme E. (1996). Micromachined Diffuser/Nozzle Elements for Valve-Less Pumps. Proc. IEEE MEMS.

[11] Olsson A., Enoksson P., Stemme G., Stemme E. (1997). Micromachined Flat-Walled Valveless Diffuser Pumps. J. Microelectromech. Syst.

[12] Olsson A., Stemme G., Stemme E. (2000). Numerical and Experimental Studies of Flat-Walled Diffuser Elements for Valveless Micropumps. Sens. Actuat. A: Phys.

[13] Jiang X.N., Zhou Z.Y., Huang X.Y., Li Y., Yang Y., Liu C.Y. (1998). Micronozzle/Diffuser Flow and Its Application in Micro Valveless Pumps. Sens. Actuat. A: Phys.

[14] Nguyen N.T., Huang X.Y. Numerical Simulation of Pulse-Width-Modulated Micropumps with Diffuser/Nozzle Elements.

[15] Pan L.S., Ng T.Y., Liu G.R., Lam K.Y., Jiang T.Y. (2001). Analytical Solutions for the Dynamic Analysis of a Valveless Micropump-a Fluid-Membrane Coupling Study. Sens. Actuat. A: Phys.

[16] Yang K.S., Chen I.Y., Shew B.Y., Wang C.C. (2003). Investigation of the Flow Characteristics within a Micronozzle/diffuser. J. Micromech. Microeng.

[17] Singhal V., Garimella S.V., Murthy J.Y. (2004). Low Reynolds Number Flow through Nozzle-Diffuser Elements in a Valveless Micropumps. Sens. Actuat. A: Phys.

[18] Ahmadian M.T., Mehrabian A. (2006). Design Optimization by Numerical Characterization of Fluid Flow through Valveless Diffuser Micropumps. J. Phys.: Conf. Ser.

[19] Nguyen N.T., Huang X. (2001). Miniature Valveless Pumps Based on Printed Circuit Board Technique. Sens. Actuat. A.: Phys.

[20] Pan L.S., Ng T.Y., Wu X.H., Lee H.P. (2003). Analysis of Valveless Micropumps with Inertial Effects. J. Micromech. Microeng.

[21] Baratti R., Cannas B., Fanni A., Pintus M., Sechi G.M., Toreno N. (2003). River Flow Forecast for Reservoir Management through Neural Networks. Neurocomputing.

[22] Ferreira P.M., Faria E.A., Ruano A.E. (2002). Neural Network Models in Greenhouse Air Temperature Prediction. Neurocomputing.

[23] Jain L.C., Vemuri V.R. (1998). Industrial Applications of Neural Networks.

[24] Goldberg D.E. (1989). Genetic Algorithms in Search, Optimization and Machine Learning.

[25] Man K.F., Tang K.S., Kwong S. (1996). Genetic Algorithms: Concepts and Applications. IEEE Trans. Industr. Electron.

[26] Arunasalam P., Seetharamu K.N., Azid I.A. (2005). Determination of Thermal Compact Model via Evolutionary Genetic Methods. IEEE Trans. Compon. Packag. Tech.

[27] Jeevan K, Quadir G.A., Seetharamu K.N., Azid I.A., Zainal Z.A. (2005). Optimization of Thermal Resistance of Stacked Micro-Channel Using Genetic Algorithms. Int. J. Numer. Meth. Heat Fluid Flow.

[28] Mitchell M. (1996). An Introduction to Genetic Algorithms.

[29] Houck C., Joines J., Kay M. (1995). A Genetic Algorithm for Function Optimization: A Matlab Implementation.

